# Contrast media and radiation dose optimization with task-based automatic keV selection: a proof-of-concept study with photon-counting detector CT

**DOI:** 10.1007/s00330-025-11738-3

**Published:** 2025-06-12

**Authors:** Konstantin Klambauer, Thomas Flohr, Lukas Jakob Moser, Victor Mergen, Matthias Eberhard, Andreas Prokein, Hatem Alkadhi, Gregor Jost, Hubertus Pietsch

**Affiliations:** 1 Diagnostic and Interventional Radiology, University Hospital Zurich, University of Zurich, Zurich, Switzerland; 2 Department of Radiology and Nuclear Medicine, Maastricht University Medical Center, Maastricht, The Netherlands; 3 Siemens Healthineers AG, Forchheim, Germany; 4 MR and CT Contrast Media Research, Bayer AG, Berlin, Germany

**Keywords:** Photon-counting detector CT, Contrast media, Radiation dose, Task-based automatic keV selection, Computed tomography angiography

## Abstract

**Objectives:**

To evaluate whether task-based automatic keV selection of photon-counting detector (PCD)-CT with optimizing radiation and contrast media (CM) dose yields consistent image quality in CT angiography (CTA).

**Materials and methods:**

PCD-CTA of the aorta was performed in six healthy minipigs across two scan sessions, with virtual monoenergetic images (VMI) reconstructed. In the first session, three protocols were conducted: the reference protocol A1 simulated standard CTA (210 mg iodine/kg CM, image quality (IQ)-level 117, non-contrast task, VMI: 70 keV); protocol A2 reduced radiation while keeping CM dose constant (210 mgI/kg, IQ-level 117, vascular task, VMI: 55 keV); and protocol A3 reduced CM dose while maintaining radiation (164 mgI/kg, IQ-level 117, non-contrast task, VMI: 55 keV). In the second session, protocols A2 and A3 were repeated as B1 and B2 to assess reproducibility, and protocol B3 further reduced the radiation dose with increased CM dose (252 mgI/kg, IQ-level 81, vascular task, VMI: 55 keV). Aortic CNR was measured; subjective assessments included contrast, noise, IQ, and visibility of intrahepatic arteries using a 4-point discrete visual scale.

**Results:**

The median CTDIvol was 3.8 mGy (A1, A3), 2.4 mGy (A2, B1), 3.9 mGy (B2), and 1.6 mGy (B3), respectively; median CM doses were 23 mL (A1, A2, B1), 18 mL (A3, B2), and 28 mL (B3), respectively. CNR was comparable across protocols (*p* = 0.906–0.947). Subjective metrics indicated diagnostic image quality (scores ≥ 2) for all protocols, with A1 and A3 having higher noise (*p* = 0.007–0.008) and lower vascular contrast (*p* = 0.003–0.008). Subjective image quality (*p* = 0.226–0.342) and visibility of intrahepatic arteries (*p* = 0.604–0.873) were similar.

**Conclusion:**

Task-based automatic keV selection enables optimization of radiation and CM dose in PCD-CTA while maintaining image quality. Protocols can be balanced to either save radiation or CM dose, depending on individual patient needs.

**Key Points:**

***Question*** *Balancing radiation and contrast media doses in CT angiography is essential, yet the full potential of photon-counting detector (PCD)CT for dose optimization remains underexplored.*

***Findings*** *Task-based automatic keV selection of PCD-CT enabled a 22% contrast reduction at constant radiation or a 58% radiation dose reduction with a compensatory 20% increase in contrast media.*

***Clinical relevance*** *Task-based automatic keV selection of PCD-CT allows individualized dose optimization by balancing radiation exposure and contrast media volume. This approach can improve patient safety by tailoring protocols to either radiation- or contrast media reduction.*

**Graphical Abstract:**

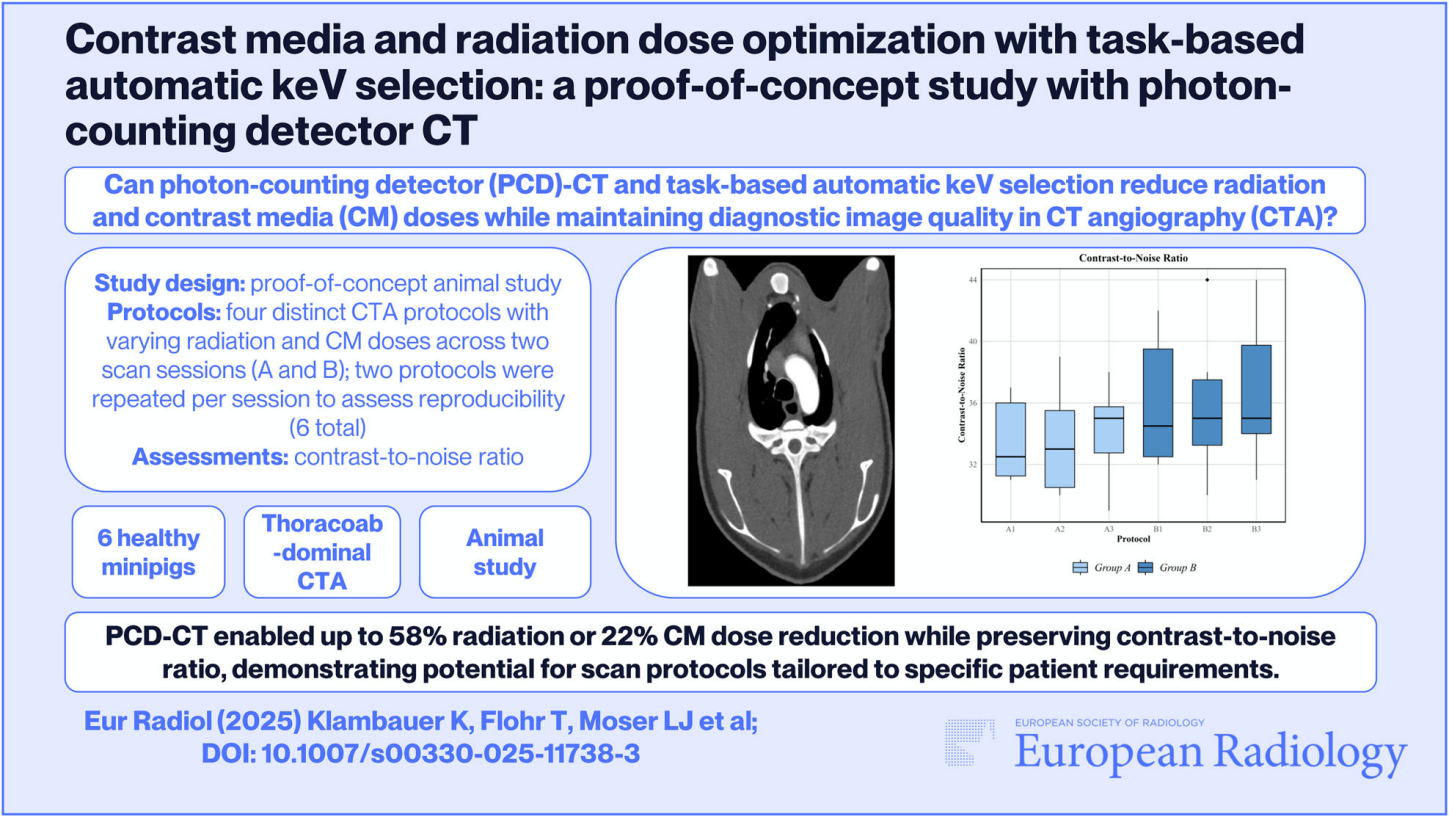

## Introduction

Developments in CT technology have focused on reducing radiation and contrast media (CM) doses. In CT angiography (CTA), image quality is largely determined by the contrast-to-noise ratio (CNR) between the vasculature and surrounding tissues, which is affected by both radiation dose and CM dose [1]. Scanning at low X-ray tube voltage has been established as a key approach to reduce radiation exposure in CTA by enhancing the iodine contrast and thus iodine CNR, which allows for a corresponding image noise increase by lowering the radiation dose [2].

Reductions in radiation dose enabled by low tube voltage scanning are sometimes implemented with concurrent reductions in CM dose, which necessitates careful optimization of imaging parameters to maintain the desired CNR [3]. By doing so, patient-specific management of radiation and CM doses for balancing image quality and safety can be performed. Elderly patients who are likely to be more sensitive to CM due to coexisting nephropathy may benefit from reduced CM dose [4, 5], while younger patients who are more likely to be vulnerable to ionizing radiation may benefit from reduced radiation doses, eventually compensated by higher CM doses to maintain the iodine CNR [6].

The issue of keV-level optimization has been evaluated with CT scanners being capable of dual-energy acquisitions with conventional energy-integrating detectors (EID), with the aim to reduce radiation and/or contrast media dose [7]. Similar to scans at low kV, VMIs at low keV values (typically 50–60 keV) significantly enhance iodine attenuation and CNR [8, 9]. In clinical routine, low tube voltage scanning can therefore be replaced by monoenergetic image reconstruction at low keV [3, 10].

Photon-counting detector (PCD)-CT further expands the concept introduced with EID-CT by providing lower noise, higher iodine CNR, and inherent spectral information with each scan [11, 12]. The first PCD-CT system in clinical use provides task-based automatic keV selection with radiation dose adjustment. Once the imaging task (vascular, parenchyma with contrast, bone, or non-contrast) and the desired image quality (IQ) level are specified, task-based automatic keV selection determines the optimal energy level for VMI reconstruction. The system then adjusts the tube current and hence, the radiation dose to maintain the desired CNR at the selected VMI energy level [13].

Prior experimental studies in phantoms with PCD-CT and task-based automatic keV selection have shown significant reductions in either radiation or CM doses [13, 14]. However, the combined strategy of reducing one parameter while compensating with another remains unexplored. Moreover, in vivo validation, currently absent in the literature, is critical due to the dynamic nature of CM distribution and kinetics, which cannot be accurately replicated using semi-anthropomorphic phantoms.

The purpose of this animal study is to evaluate whether task-based automatic keV selection with optimizing radiation and CM dose yields consistent CNR in PCD-CTA.

## Materials and methods

### Subjects

The study was performed on six healthy Göttingen minipigs (Ellegaard) with a median body weight of 32.5 kg (Q1, Q3: 29.8–34.6 kg) and a median transverse abdominal cross-section of 23.3 cm (Q1, Q3: 20.9–25.7 cm). Animals were handled according to the National Animal Welfare Legislation, with approval from the state animal welfare committee. CT scans were conducted under general anesthesia, initiated with an intramuscular injection of 15 mg/kg ketamine (Pharmacia), 2 mg/kg azaperone (Stresnil, Elanco GmbH), and 0.02 mg/kg atropine (Eifelfango Chem.-Pharm. Werke). Following an intravenous dose of 7 mg/kg propofol (Propofol-Lipuro, Braun), the animals were orally intubated and ventilated with an air/oxygen mixture. Anesthesia was maintained with 9–12 mg/kg/h propofol. CTA was performed with the animals in the prone position during end-expiratory ventilation hold, while heart rate and oxygen saturation were monitored. The same minipigs underwent CTA with low tube voltage in another, separate scan session [15].

### Data acquisition

Scans were performed using a first-generation, dual-source PCD-CT (NAEOTOM Alpha, version VB10A, Siemens Healthineers AG) equipped with two cadmium-telluride PCDs. Scan data were acquired in the single-source QuantumPlus mode, providing spectral information, with a detector collimation of 144 × 0.4 mm, a gantry rotation time of 0.25 ms, a pitch factor of 0.8, and a tube voltage of 140 kV for all protocols. Images were reconstructed with a median-sharp Qr40 kernel, quantum iterative reconstruction (QIR) level 3, a field of view (FOV) of 300 × 300 mm², slice thickness of 0.8 mm, and increment of 0.6 mm. Both task-based automatic keV selection (CARE keV, Siemens) and automated anatomical tube current modulation (CARE Dose 4D, Siemens) were used.

### Theoretical background

Based on a standard PCD-CTA protocol, our study aimed to develop protocols that either achieve a reduction in radiation dose *D*_*R*_ at constant or even increased CM dose *D*_*C*_, or a reduction in CM dose *D*_*C*_ at constant radiation dose *D*_*R*_. The following well-known correlations were used for the adjustments of *D*_*R*_, *D*_*C*_, and the expected resulting CNR described below:

The CNR is proportional to the square root of the radiation dose $\sqrt{{D}_{R}}$, because the image noise σ is expected to be proportional to $1/\sqrt{{D}_{R}}$. If CNR_1_ is obtained at a radiation dose *D*_*R1*_, changing the radiation dose to *D*_*R2*_ results in

1 $${{{{\rm{CNR}}}}}_{2}=\sqrt{{D}_{R2}/{D}_{R1}}{{{{\rm{CNR}}}}}_{1}$$

The CNR is expected to be proportional to the CM dose *D*_*C*._ for small variations of a standard contrast protocol. If CNR_1_ is obtained at a CM dose *D*_*C1*_, changing the CM dose to *D*_*C2*_ results in

2 $${{{{\rm{CNR}}}}}_{2}={D}_{C2}/{D}_{C1}{{{{\rm{CNR}}}}}_{1}$$

As a consequence of (1) and (2), changing the radiation dose from *D*_*R1*_ to *D*_*R2*_ must be compensated by a corresponding change in the CM dose to maintain the CNR

3 $${{{D}}}_{{{C}}2}=\sqrt{{D}_{R1}/{D}_{R2}}{{{{\rm{D}}}}}_{{{{\rm{C}}}}1}$$

### Study design

The study evaluated four distinct CTA protocols across two scan sessions (Groups A and B), each varying in radiation and CM doses. Three protocols were performed per session: protocols A1–A3 in the first session, and protocols B1–B3 in the second session. Protocols A2 and A3 were repeated as B1 and B2, respectively, to assess reproducibility under comparable conditions. The four protocols included a reference protocol (A1) with standard radiation and CM dose, a protocol with reduced radiation dose and constant CM dose (A2/B1), a protocol with reduced CM dose and constant radiation dose (A3/B2), and a protocol with further radiation dose reduction compensated by increased CM dose (B3). All six minipigs underwent both scan sessions, receiving all protocols in a randomized order within each session. While the ideal design would have applied all protocols in a single session per animal, the local animal welfare committee restricted anesthesia duration, necessitating the division into two sessions. This approach allowed full protocol testing and reproducibility assessment while adhering to ethical standards. To minimize potential bias, a 45-min CM washout period was observed between scans, and topograms were not repeated between protocols to avoid unintended effects on automated tube current modulation. The urinary bladder was excluded from the scan range to prevent artifacts from excreted CM. Radiation dose was assessed using the volume CT dose index (CTDIvol) for each scan. Protocols are detailed as follows (Table 1):

- **A1: Reference**

**Table 1:** Technical parameters of protocols

Protocol	Task-based automatic keV selection (CARE keV)	IQ-level	Reconstruction energy (keV)	Contrast media dose (mgI/kg)	Contrast media flow (mL/s)	Iodine flux (gI/s)
A1	Non-contrast/70	117	70	210	3.5	0.735
A2/B1	Vascular/55	117	55	210	3.5	0.735
A3/B2	Non-contrast/70	117	55	164	2.7	0.442
B3	Vascular/55	81	55	252	4.2	1.058

keV values represent the optimal reconstruction energy for imaging tasks in CARE keV and the actual reconstruction energy of virtual monoenergetic images (VMI) after image acquisition. Protocol A1 was set as reference

*IQ* image quality

- IQ-level set at 117, representing the default level from the vendor for an abdominal CTA scan, with CARE keV optimized for non-contrast (70 keV) and reconstruction of VMIs at 70 keV. The iodine CNR in VMIs at 70 keV equals conventional spectral CT images acquired at a tube voltage of 120 kV (10). Protocol A1 therefore corresponds to a conventional CTA of the aorta at 120 kV and served as the reference protocol in terms of radiation and CM dose: *D*_*R,A1*_ = 100%, *D*_*C,A1*_ = 100%.

- **A2: Reduced radiation dose**

- IQ-level set at 117 with CARE keV optimized for vascular (55 keV) and reconstruction of VMIs at 55 keV. Change of the CARE kV imaging task to “vascular” automatically reduces the radiation dose to 61%: *D*_*R,A2*_ = 0.61_*DR,A1*_ [13]. Iodine CNR is expected to be maintained because of VMI reconstructions at 55 keV instead of 70 keV as in A1, and the contrast protocol is left unchanged. A2 corresponds to a state-of-the-art low keV CTA protocol of the aorta, with reduced radiation dose while maintaining CM dose compared to the reference A1: *D*_*R,A2*_ = 61%, *D*_*C,A2*_ = 100%.

- **A3: Reduced CM dose**

- IQ-level set at 117 with CARE keV optimized for non-contrast as in A1 (70 keV), but with manual override of the suggested keV level and reconstruction of VMIs at 55 keV as in A2. The radiation dose therefore corresponds to the reference dose of protocol A1: *D*_*R,A3*_ = *D*_*R,A1*_ (100%). The iodine CNR, however, is increased because of VMI reconstructions at 55 keV. According to (1), the iodine CNR is expected to be increased to ${{{{\rm{CNR}}}}}_{{{{\rm{A}}}}3}=\sqrt{{D}_{R,A3}/{D}_{R,A2}}{{{{\rm{CNR}}}}}_{{{{\rm{A}}}}2}=\sqrt{{D}_{R,A1}/0.61{D}_{R,A1}}{{{{\rm{CNR}}}}}_{{{{\rm{A}}}}2}=1.28\,{{{{\rm{CNR}}}}}_{{{{\rm{A}}}}2}$ [16]. With (3), we expect a possible CM dose reduction to ${D}_{C,A3}=\sqrt{{D}_{R,A2}/{D}_{R,A3}}{D}_{C,A2}=\sqrt{0.61{D}_{R,A1}/{D}_{R,A1}}{D}_{C,A2}=$0.78$\,{D}_{C,A2}$ in order to maintain the CNR of A2. Unlike Protocol A3, protocol A3 modifies the CARE keV mechanism to achieve a reduction in CM dose (78% of the reference) while maintaining the same radiation dose as the reference protocol A1 (100%): *D*_*R,A3*_ = 100%, *D*_*C,A3*_ = 78%.

- **B1: Reduced radiation dose**

- Protocol A2 was repeated with the same scan and CM protocol to test the reproducibility.

- **B2: Reduced CM dose**

- Protocol A3 was repeated with the same scan and CM protocol to test the reproducibility.

- **B3: Further reduction of radiation dose**

- IQ-level set at 81 with CARE keV optimized for vascular (55 keV) and reconstruction of VMIs at 55 keV (same as A2). Radiation dose was reduced to *D*_*R,B3*_ = 81/117*D*_*R,A2*_ = 0.69 *D*_*R,A2*_ = 0.42 *D*_*R,A1*_ compared to the reference A1 by lowering the IQ-level from 117 to 81. According to (3), the expected lower CNR compared to A2 must be compensated for by increasing the CM dose to ${D}_{C,B3}=\sqrt{{D}_{R,A2}/{D}_{R,B3}}{D}_{C,A2}=\sqrt{{D}_{R,A2}/{0.69D}_{R,A2}}{D}_{C,A2}$=1.2$\,{D}_{C,A2}$. Protocol B3 enables a more significant radiation dose reduction than A2 by accepting a higher CM dose: *D*_*R,B3*_ = 42%, *D*_*C,B3*_ = 120%.

### Contrast protocol

**Fig. 1:**
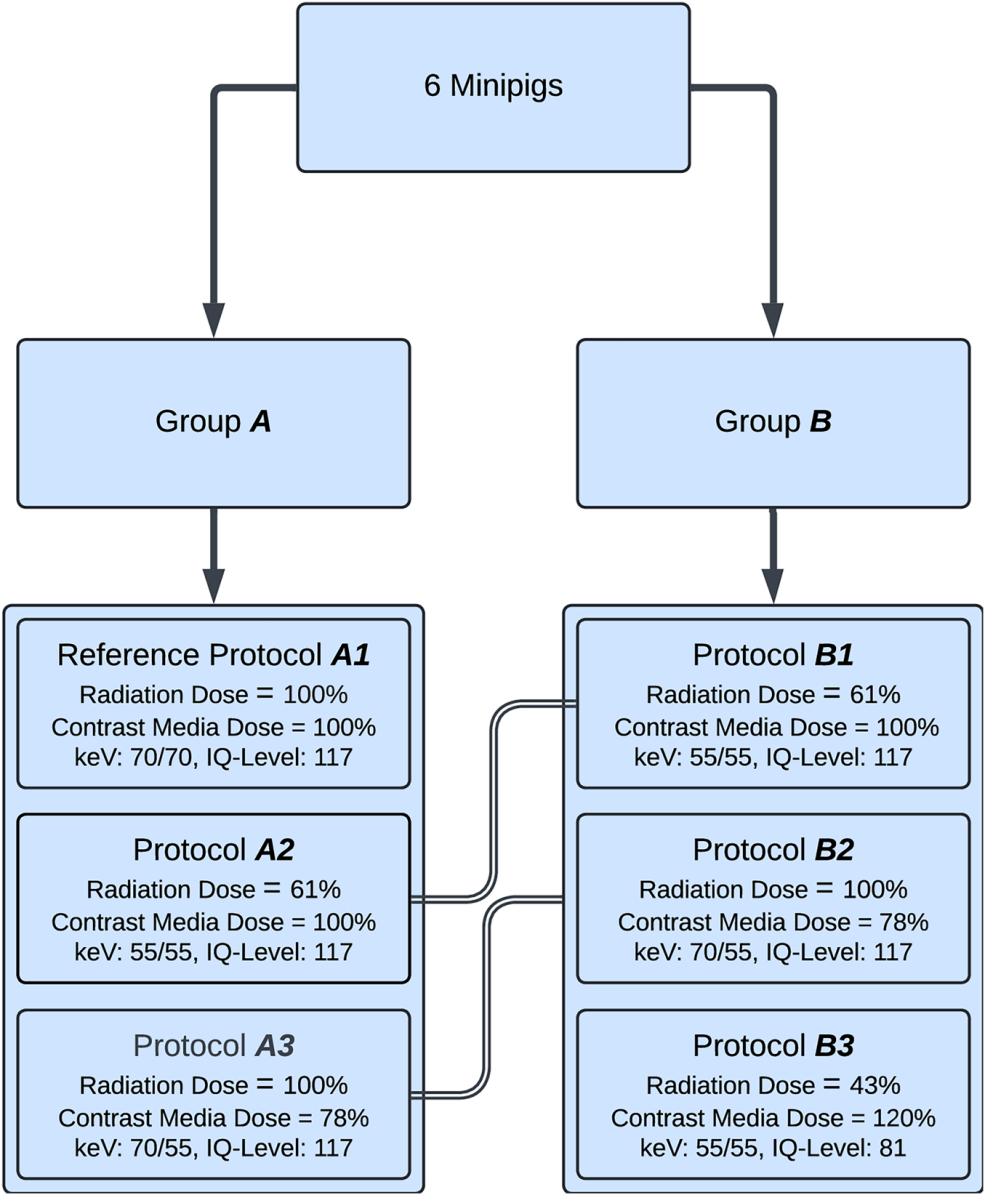
Flowchart of the study. Protocol A2 = B1, A3 = B2. IQ, image quality; VMI, virtual monoenergetic images (selected reconstruction energy in CARE keV/ reconstruction energy for VMI in keV)

**Fig. 2:**
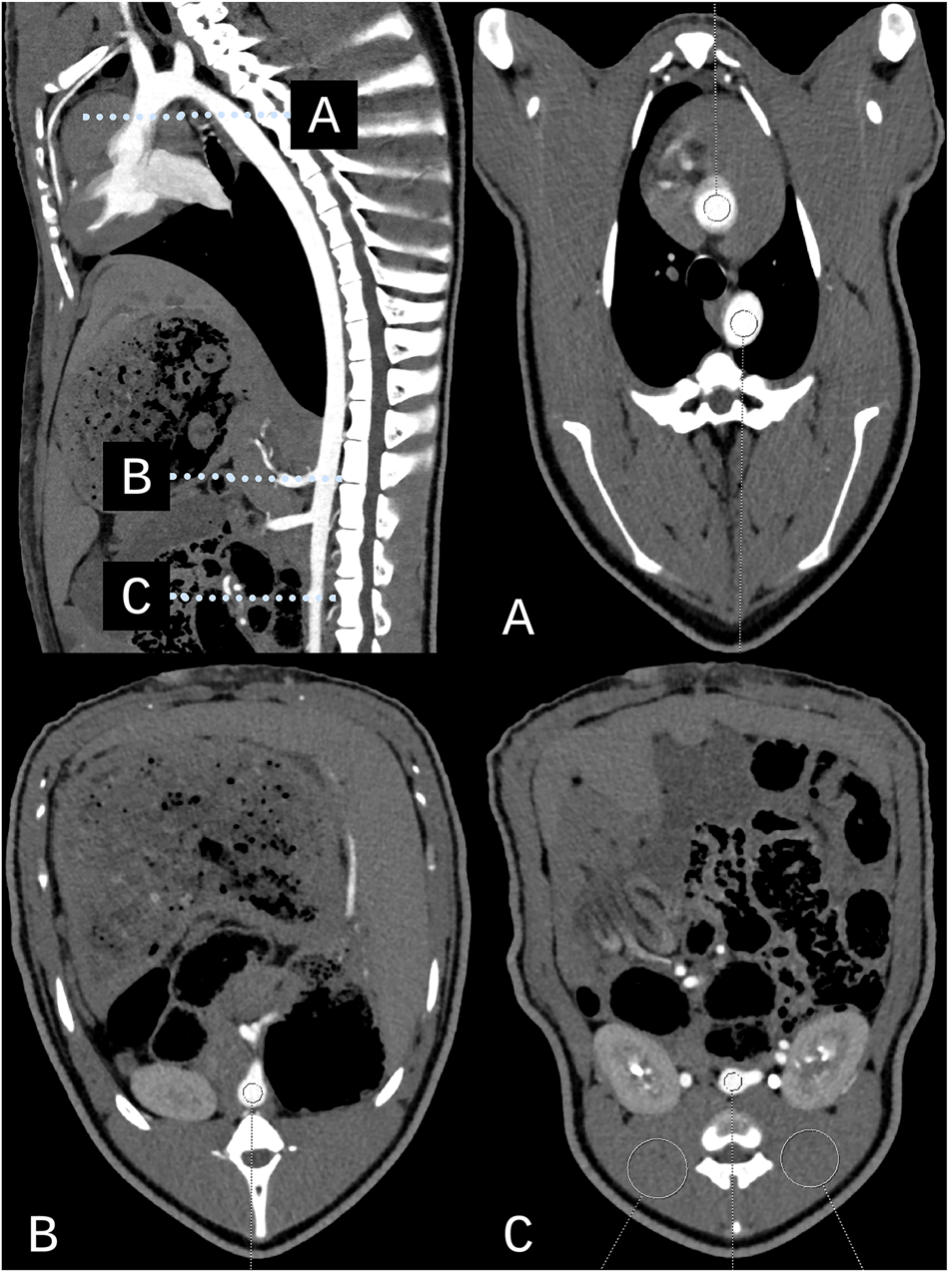
ROI placement in aorta and back muscle. Top left: Sagittal images of the minipigs‘ thoracic and abdominal aorta showing different aortic levels. ROI placement was performed on axial images at the following levels: **A** ascending and descending aorta, (**B**) abdominal aorta at the level of the celiac trunk, and (**C**) abdominal aorta at the level of the renal arteries, with additional bilateral ROIs placed in the back muscles

Iopromide 300 mgI/mL (Ultravist 300, Bayer Vital GmbH) was administered in a body weight-adapted dosage, followed by a 20 mL saline flush using a power injection system (Medrad Centargo, Bayer AG). The CM flow rate was adjusted according to the administered CM dose to ensure identical bolus lengths across all CTA protocols. Bolus tracking (90 kV, cycle time 0.8 s) was performed in the descending aorta (trigger level = 100 HU, trigger delay = 3 s). CM volume (mL) for each scan was recorded. Figures 1, 2 and Table 1 outline the study design with the different scan protocols used.

### Objective image assessment

Attenuation *A* (in HU) was measured in four circular regions of interest (ROIs) in the ascending aorta, descending aorta, abdominal aorta at the level of the celiac trunk, and at the level of the renal arteries. Additionally, attenuation and image noise were measured bilaterally in circular ROIs placed in the deep back muscles at the level of the renal arteries. Image noise σ (in HU) was measured as the standard deviation of attenuation in the ROI. Figure 2 visualizes the different levels of aortic and muscular ROI placements. The CNR was then calculated using the mean of all aortic and muscular measurements according to:

4 $${CNR}=\frac{{A}_{{aorta}}-{A}_{{muscle}}}{{\sigma }_{{muscle}}}$$

### Subjective image assessment

Two independent radiologists (K.K. and L.M., with 7 and 4 years of experience in cardiovascular CT) evaluated the blinded CT scans by rating four aspects on a 4-point discrete visual scale: overall image quality, vascular contrast, noise, and visibility of the distal hepatic arteries. The scale was defined as follows: 1 = non-diagnostic, 2 = moderate but diagnostic, 3 = good, and 4 = excellent. The overall image quality and visibility of distal hepatic arteries were assessed with strategic windowing to maintain a stable, perceived visual CNR. As a wider window width reduces perceived noise [17], we used the following formula for window width selection (a window level of 150 was chosen for all protocols):

$${window\; width}={{{\rm{Image\; noise}}}}\,{{{\rm{\sigma }}}}* 50$$

This factor was determined at readouts to be the best trade-off between image contrast and noise.

### Statistical analyses

Results were presented using median (Q1, Q3) or mean (standard deviation) based on data distribution, assessed with the Shapiro–Wilk test. Due to non-normality, Mann–Whitney U with Bonferroni correction was used for pairwise comparisons, and Kruskal–Wallis for group differences. Medians with interquartile ranges were visualized with boxplots. Inter- and intrareader reliability for subjective image quality assessment was evaluated with Cohen’s squared kappa statistic (< 0.20, no; 0.20–0.39, minimal; 0.40–0.59, weak; 0.60–0.79, moderate; 0.80–0.89 strong; 0.89–1.00, almost perfect) [18, 19]. Statistical significance was set at *p* < 0.05. Analyses were performed using open-source statistical software R (version 4.4.0, R Core Team).

## Results

### Radiation dose and contrast media volume

Protocols A2/B1 and A3/B2 demonstrated comparable results without significant differences in radiation dose and CM volume (*p* > 0.999). The reference protocol A1 for both radiation dose and CM volume had a CTDI_vol_ of 3.8 mGy and a CM volume of 23 mL. In Protocol A3, the radiation dose remained identical to A1 (CTDI_vol_ 3.8 mGy), while the CM volume was reduced to 18 mL (78% of A1). Protocol A2 had a reduced radiation dose (CTDI_vol_ 2.4 mGy, 63% of A1) with the CM volume unchanged at 23 mL (100% of A1). Protocol B3 exhibited the lowest radiation dose (CTDI_vol_ 1.6 mGy, 42% of A1) and an increased CM volume of 28 mL (122% of A1). Table 2 details the radiation and CM doses of each protocol.

**Table 2:** Radiation dose and contrast volume of study protocols

Protocol	Tube current-time product (mAs)	Radiation dose - CTDI_vol_ (mGy)	Relative radiation dose (%)	Contrast media volume (mL)	Relative contrast media volume (%)
A1	33 (31, 35)	3.8 (3.6, 4.0)	100	23 (21, 24)	100
A2	21 (19, 22)	2.4 (2.3, 2.5)	63	23 (21, 24)	100
A3	33 (31, 35)	3.8 (3.6, 4.0)	100	18 (16, 19)	78
B1	21 (20, 23)	2.4 (2.2, 2.5)	63	23 (21, 24)	100
B2	34 (32, 36)	3.9 (3.7, 4.1)	102	18 (16, 19)	78
B3	15 (13, 15)	1.6 (1.5, 1.8)	42	28 (25, 29)	122

Values are provided in median (Q1, Q3). B1/2/3 and A1/A2: *p* < 0.001 for tube current and radiation dose measurements; others were not significant. Protocol A1 was set as reference for percentage calculation. CTDI_vol_ was used

*CTDI*_*vol*_ volume CT dose index, *CM* contrast media

### Quantitative image assessment

In protocols A, aortic attenuation showed significant differences across protocols (*p* < 0.001). A2 had the highest median attenuation (772 HU), followed by A3 (574 HU) and A1 (421 HU). Noise also varied significantly (*p* < 0.001), with A2 showing the highest noise (20 HU), followed by A3 (16 HU) and A1 (10 HU). CNR remained constant, with no differences across protocols (*p* = 0.906).

**Fig. 3:**
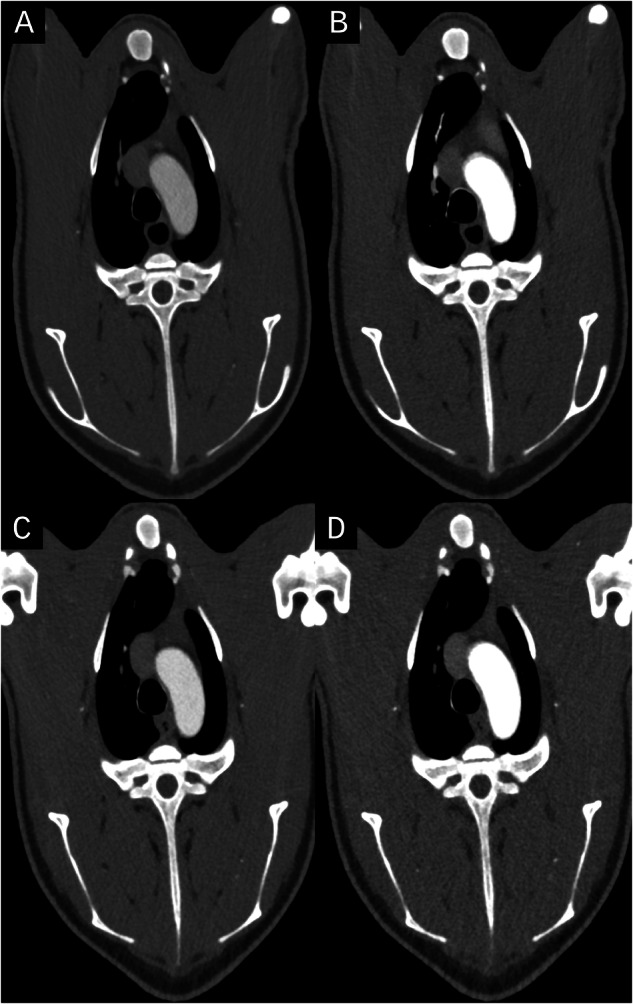
Axial images of the four distinct scan protocols tested per minipig. **A** Protocol A1, **B** Protocol A2/B1, **C** Protocol A3/B2, **D** Protocol B3. Images are shown with the same window level and width before strategic windowing was performed for subjective image assessment

In protocols B, aortic attenuation was significantly higher in B3 (935 HU) compared to B1 (744 HU) and B2 (565 HU) (*p* = 0.002). Noise also differed significantly (*p* = 0.002), with B3 showing the highest noise (22 HU), followed by B1 (18 HU) and B2 (14 HU). CNR did not differ between protocols (*p* = 0.947). Protocols A2/B1 and A3/B2 showed nearly identical results in median attenuation, noise and CNR (*p* = 0.265–0.999) (Table 3, Supplementary Table, Fig. 3).

**Table 3:** Groupwise comparisons for quantitative and qualitative image assessments

Parameters for protocols A	A1	A2	A3	*p*-value
Attenuation aorta (HU)	421 (401, 442)	772 (698, 784)	574 (564, 615)	< 0.001
Attenuation muscle (HU)	70 (69, 70)	70 (69, 72)	70 (69, 72)	0.456
Noise muscle (HU)	10 (10, 11)	20 (20, 21)	16 (14, 16)	< 0.001
Contrast-to-noise ratio	32 (31, 36)	33 (30, 36)	35 (33, 36)	0.906
Subjective image quality (1–4)	4 (3, 4)	3 (3, 3)	4 (3, 4)	0.226
Subjective contrast (1–4)	2 (2, 3)	4 (4, 4)	3 (3, 3)	0.003
Subjective noise (1–4)	4 (4, 4)	3 (2, 3)	4 (3, 4)	0.008
Subjective visibility of intrahepatic arteries (1–4)	3 (3, 3)	3 (3, 3)	4 (3, 4)	0.604

Parameters for protocols B	B1	B2	B3	*p*-value
Attenuation aorta (HU)	744 (699, 764)	565 (513, 636)	935 (839, 960)	0.002
Attenuation muscle (HU)	71 (70, 71)	68 (67, 70)	72 (70, 74)	0.153
Noise muscle (HU)	18 (17, 19)	14 (13, 15)	22 (21, 24)	0.002
Contrast-to-noise ratio	34 (32, 40)	35 (33, 38)	35 (34, 40)	0.947
Subjective image quality (1–4)	3 (3, 4)	4 (3, 4)	3 (3, 3)	0.342
Subjective contrast (1–4)	4 (3, 4)	3 (3, 3)	4 (4, 4)	0.008
Subjective noise (1–4)	3 (3, 3)	4 (3, 4)	2 (2, 3)	0.007
Subjective visibility of intrahepatic arteries (1–4)	3 (3, 4)	3 (3, 4)	4 (2, 4)	0.873

Values are provided in median (Q1, Q3). *p*-values after groupwise Kruskal–Wallis test

*HU* Hounsfield units

### Subjective image assessment

In Group A, subjective vascular contrast varied significantly across protocols (*p* = 0.003). A2 received the highest ratings (4 [Q1, Q3: 4, 4]), while A1 was rated lower (2 [Q1, Q3: 2, 3]). Subjective noise also differed significantly (*p* = 0.008), with A1 rated best (4 [Q1, Q3: 4, 4]) and A2 rated worst (3 [Q1, Q3: 2, 3]). There were no significant differences in subjective image quality (*p* = 0.226) or subjective visibility of intrahepatic arteries (*p* = 0.604).

In Group B, subjective vascular contrast was significantly higher in B3 (4 [Q1, Q3: 4, 4]) compared to B1 and B2 (*p* = 0.008). Subjective noise showed significant differences (*p* = 0.007), with B3 rated worst (2 [Q1, Q3: 2, 3]). There were no significant differences in subjective image quality (*p* = 0.342) or subjective visibility of intrahepatic arteries (*p* = 0.873). A2/B1 and A3/B2 also yielded similar results in the qualitative assessments (Table 3, Supplementary Table).

**Fig. 4:**
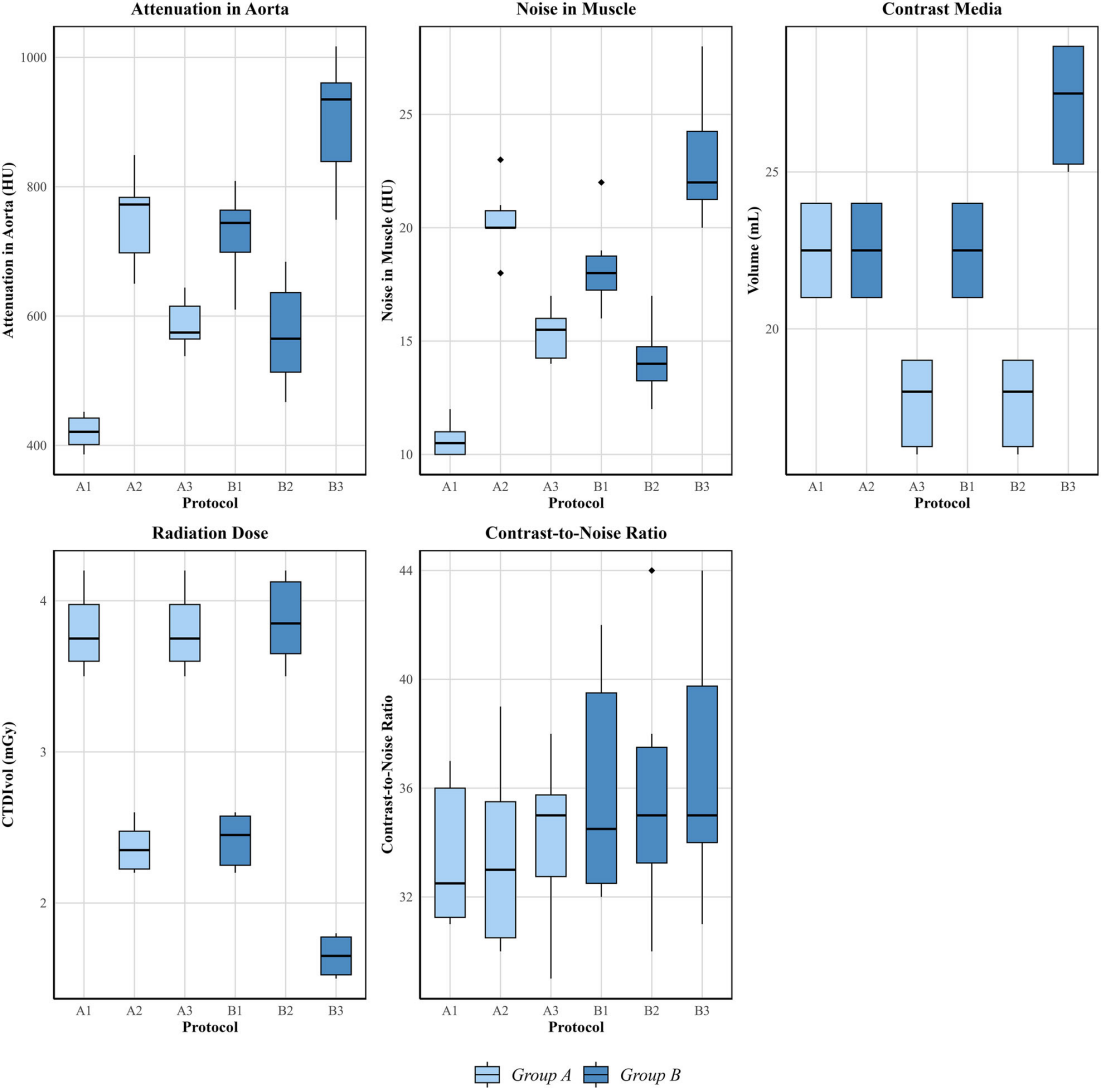
Boxplots for attenuation, noise, contrast-to-noise ratio, radiation dose, and contrast media volume. CTDI_vol_, volume CT dose index

For both groups, all protocols yielded diagnostic (≥ 2) scores across all qualitative assessment categories (Fig. 4). Intrareader reliability was almost perfect (Cohen’s kappa: 0.867, *p* < 0.001), and interreader reliability was substantial (Cohen’s kappa: 0.795, *p* < 0.001).

## Discussion

No studies so far have investigated optimization strategies for PCD-CTA protocols in which reducing one parameter (e.g., radiation dose) necessitates compensatory adjustments in another (e.g., contrast media dose), or vice versa. This proof-of-concept study in animals aimed to address this knowledge gap for providing a foundation for future human trials focusing on personalized dose optimization. By combining task-based automatic keV selection with radiation dose adjustment, adaptation of IQ-level and CM dose, and specific selection of the VMI reconstruction energy, the main study findings were as follows:

(1) Radiation doses could be reduced by up to 58% and CM dose by 22%, with the largest radiation dose reductions possible when simultaneously increasing the CM dose; and (2) subjective image quality including the visibility of small intrahepatic arteries was constantly high across protocols, rated between good and excellent.

Previous studies evaluated strategies for CM and radiation dose reduction with EID-CT scanners being capable of dual-energy acquisitions [20, 21]. As opposed to EID-CT, PCD-CT offers the absence of electronic noise, lower image noise, higher iodine CNR, and more inherent spectral imaging, which altogether may enable greater flexibility in individualized protocol optimization [7]. Our study confirms the feasibility of achieving similar or greater dose reductions with PCD-CT. PCD-CT has demonstrated improved iodine CNR compared to EID-CT [22], with studies reporting up to 38% higher iodine CNR, allowing for reductions in radiation and/or CM dose [11, 12, 23]. Minimizing radiation dose is particularly beneficial for younger patients, while reducing CM dose may mitigate nephrotoxicity risk in older or renally impaired individuals [4–6].

Rajendran et al evaluated task-based automatic keV selection and radiation dose adjustments in PCD-CT of torso-shaped phantoms and demonstrated radiation dose reductions of 9%, 21%, and 39% for bone, parenchyma, and vascular imaging tasks, respectively, compared to non-contrast imaging [13]. Our study further expands on this concept by demonstrating a 37% radiation dose reduction in vascular imaging with a fixed tube voltage and task-based keV-dependent radiation dose adjustments while keeping CM dose constant. Importantly, an even further 58% reduction in radiation dose was possible while maintaining a constant CNR, at the same time increasing the CM dose by 22%.

In a phantom PCD-CT study by Emrich et al, diagnostic CNR was maintained in coronary CTA with CM dose reductions of up to 50% using 40 keV VMIs, or 25% with 65 keV VMIs [14]. Cundari et al demonstrated diagnostic image quality in coronary CTA in humans with a 40% CM dose reduction using 45 keV VMIs [24]. Our animal study on CTA achieved similar CNR with a 22% CM dose reduction at 55 keV VMIs compared to 70 keV VMIs. These findings underscore the potential of PCD-CT to reduce CM doses without compromising image quality.

Using EID-CT with automatic tube voltage selection, Haubold et al achieved 26% CM and 30% radiation dose reductions relative to a 120 kV reference protocol [25]. In contrast, our PCD-CT study achieved comparable CM dose reductions (22%) but substantially higher radiation dose reductions (58%), while preserving the full spectral capabilities of PCD-CT. Notably, Haubold et al reported a slight decline in diagnostic acceptability in radiation-saving protocols [25], an issue that did not arise in our study.

The following study limitations must be acknowledged. First, as with all animal studies, there are inherent limitations in translating findings directly to clinical practice in patients due to physiological differences such as size and weight. Nonetheless, the abdominal cross-section of the pigs (median 23.3 cm) was only slightly smaller than the range typically reported in human abdominal imaging (25–42 cm) [26], supporting the relevance of this model for CTA protocol development. Second, the study was limited to six animals, which reduces the statistical power, particularly for subjective image quality assessments. Third, although all four protocols were tested in every animal, they were distributed across two scan sessions due to restrictions imposed by regulations from the local animal welfare committee on maximum anesthesia time. This hindered testing all protocols within a single session in each animal. To mitigate this limitation, reproducibility was tested by repeating two of the protocols in a second scan session. Fourth, only one protocol focused on contrast media dose reduction, limiting the generalization of further CM-saving strategies. Lastly, since all animals were healthy, this study did not evaluate diagnostic performance, which would require pathological findings and clinical correlation.

In conclusion, this proof-of-concept study in healthy animals demonstrated that PCD-CT with task-based automatic keV selection, including radiation dose adjustments and optimal VMI energy level selection, can reduce radiation doses by 58% and CM doses by 22% while maintaining stable CNR and diagnostic image quality through strategic dose adjustments. Our results thus highlight the potential of PCD-CT for optimizing dose tailored to specific clinical needs. Further validation in humans is required to demonstrate the clinical applicability and effectiveness of these optimized CTA protocols.

## Supplementary information

ELECTRONIC SUPPLEMENTARY MATERIAL

## Abbreviations

CM: Contrast media
CNR: Contrast-to-noise ratio
CTDI: Computed tomography dose index
EID-CT: Energy integrating detector computed tomography
HU: Hounsfield units
IQ: Image quality
PCD-CT: Photon counting detector computed tomography
VMI: Virtual monoenergetic image
