# Supplementary material for: Contrast media and radiation dose optimization with task-based automatic keV selection: a proof-of-concept study with photon-counting detector CT
Source: Eur Radiol. 2025 Jun 12;35(12):7975–84. doi: 10.1007/s00330-025-11738-3 (PMC12634813; doi:10.1007/s00330-025-11738-3)
Supplement: Supplementary file 1 — ELECTRONIC SUPPLEMENTARY MATERIAL [file 330_2025_11738_MOESM1_ESM.pdf]

Contrast media and radiation dose optimization with task-based automatic keV selection: A proof-of-concept study with photon-counting detector CT

Electronic Supplementary Material (ESM)

Eur Radiol (2025) Klambauer K, Flohr T, Moser LJ et al.

Supplement

Supplemental Table. *Pairwise comparison of protocols*

Parameters for protocols A	A1 vs. A2	A1 vs. A3	A2 vs. A3
Attenuation Aorta (HU)	0.016	0.016	0.016
Attenuation Muscle (HU)	0.749	<0.999	<0.999
Noise Muscle (HU)	0.014	0.014	0.014
Contrast-to-Noise Ratio	<0.999	<0.999	<0.999
Subjective Image Quality (1-4)	0.848	<0.999	0.337
Subjective Vascular Contrast (1-4)	0.015	0.068	0.103
Subjective Noise (1-4)	0.022	<0.999	0.055
Subjective Visibility of Intrahepatic Arteries (1-4)	<0.999	<0.999	<0.999
**Parameters for protocols B**	**B1 vs. B2**	**B1 vs. B3**	**B2 vs. B3**
Attenuation Aorta (HU)	0.059	0.040	0.015
Attenuation Muscle (HU)	<0.999	<0.999	0.156
Noise Muscle (HU)	0.030	0.048	0.015
Contrast-to-Noise Ratio	<0.999	<0.999	<0.999
Subjective Image Quality (1-4)	<0.999	<0.999	0.678
Subjective Vascular Contrast (1-4)	0.272	0.522	0.025
Subjective Noise (1-4)	0.163	0.337	0.040
Subjective Visibility of Intrahepatic Arteries (1-4)	<0.999	<0.999	<0.999
**Parameters for protocols A and B**	**A1 vs. B3**	**A2 vs. B1**	**A3 vs. B2**
Attenuation Aorta (HU)	0.015	<0.999	<0.999
Attenuation Muscle (HU)	0.186	<0.999	<0.999
Noise Muscle (HU)	0.014	0.265	0.657
Contrast-to-Noise Ratio	0.879	0.995	<0.999
Subjective Image Quality (1-4)	0.604	<0.999	<0.999
Subjective Vascular Contrast (1-4)	0.007	<0.999	<0.999
Subjective Noise (1-4)	0.015	<0.999	<0.999
Subjective Visibility of Intrahepatic Arteries (1-4)	<0.999	<0.999	<0.999

Note. P-values after pairwise Mann-Whitney-U test and Bonferroni-correction for multiple testing.
HU=Hounsfield Units
